# Ethylene and reactive oxygen species are involved in root aerenchyma formation and adaptation of wheat seedlings to oxygen-deficient conditions

**DOI:** 10.1093/jxb/ert371

**Published:** 2013-11-19

**Authors:** Takaki Yamauchi, Kohtaro Watanabe, Aya Fukazawa, Hitoshi Mori, Fumitaka Abe, Kentaro Kawaguchi, Atsushi Oyanagi, Mikio Nakazono

**Affiliations:** ^1^Graduate School of Bioagricultural Sciences, Nagoya University, Furo-cho, Chikusa, Nagoya 464-8601, Japan; ^2^NARO Institute of Crop Science, 2-1-18, Kannondai, Tsukuba, Ibaraki 305-8518, Japan

**Keywords:** Aerenchyma, ethylene, fermentation, oxygen deficiency, reactive oxygen species, wheat (*Triticum aestivum L.*).

## Abstract

Exposing plants to hypoxic conditions greatly improves their anoxic stress tolerance by enhancing the activities of glycolysis and fermentation in roots. Ethylene may also be involved in these adaptive responses because its synthesis is increased in roots under hypoxic conditions. Here it is reported that pre-treatment of wheat seedlings with an ethylene precursor, 1-aminocyclopropanecarboxylic acid (ACC), enhanced accumulation of ethylene in the roots of wheat seedlings, and enhanced their tolerance of oxygen-deficient conditions through increasing the expression of genes encoding ethanol fermentation enzymes, alcohol dehydrogenase and pyruvate decarboxylase, in the roots. Lysigenous aerenchyma formation in root was induced by ACC pre-treatment and was further induced by growth under oxygen-deficient conditions. ACC pre-treatment increased the expression of three genes encoding respiratory burst oxidase homologue (a plant homologue of gp91^phox^ in NADPH oxidase), which has a role in the generation of reactive oxygen species (ROS), in roots of seedlings. Co-treatment with ACC and an NADPH oxidase inhibitor, diphenyleneiodonium, partly suppressed the ACC-induced responses. These results suggest that ethylene and ROS are involved in adaptation of wheat seedlings to oxygen-deficient conditions through controlling lysigenous aerenchyma formation and the expression of genes encoding ethanol fermentation enzymes.

## Abstract (precis)

Ethylene-mediated reactive oxygen species signalling is involved in adaptive responses of wheat seedlings to waterlogged conditions through controlling formation of lysigenous aerenchyma and expression of genes encoding ethanol fermentation enzymes in roots

## Introduction

The principal cause of damage to plants grown in waterlogged soil is inadequate supply of oxygen to the submerged tissues (Blom, 1999; Colmer and Voesenek, 2009). Oxygen deficiency in roots occurs with poor drainage after rain or irrigation, causing depressed growth of dryland plant species (Drew, 1997). Under natural conditions, roots are exposed to a gradual transition from normoxia to hypoxia, providing an opportunity for acclimation before conditions become anoxic (Drew, 1997). Exposing plants to hypoxic conditions greatly improves anoxic stress tolerance (Waters *et al.*, 1991; Dolferus *et al.*, 1997; Germain *et al.*, 1997; Ellis and Setter, 1999). Exposure of roots to hypoxic conditions substantially increased the activities of two ethanol fermentation enzymes, alcohol dehydrogenase (ADH) and pyruvate decarboxylase (PDC), in wheat (*Triticum aestivum* L.; Waters *et al.*, 1991), maize (*Zea mays* L.; Wignarajah and Greenway, 1976), and barley (*Hordeum vulgare* L.; Wignarajah *et al.*, 1976). Transient induction of *ADH1* transcripts and only a small induction of ADH enzyme activity were observed in root tips of aerobically grown maize seedlings subjected to anoxic conditions (Andrews *et al.*, 1993), whereas acclimation of root tips of seedlings by hypoxic pre-treatment enhanced induction of both *ADH1* mRNA and ADH enzyme activity under subsequent anoxic conditions (Andrews *et al.*, 1994*a*). Hypoxia also increased the levels of *PDC* transcripts in root tips of maize seedlings (Andrews *et al.*, 1994*b*).

Internal transport of oxygen from shoots to roots is essential to survival and functioning of roots (Armstrong, 1979). To adapt to waterlogging in soil, some gramineous plants develop lysigenous aerenchyma (Arikado and Adachi, 1955; Trought and Drew, 1980*a*; Drew *et al.*, 1981; Jackson *et al.*, 1985*b*), which is formed by the creation of gas spaces as a result of death and the subsequent lysis of some cells, in the root cortex (Jackson and Armstrong, 1999; Evans, 2003). In some wetland plants, root lysigenous aerenchyma is constitutively formed under drained soil conditions (Jackson *et al.*, 1985*b*; Visser and Bögemann, 2006; Abiko *et al.*, 2012; Mano and Omori, 2013), and its formation is enhanced upon soil waterlogging (Colmer *et al.*, 2006; Shiono *et al.*, 2011). On the other hand, in dryland plants (e.g. wheat), lysigenous aerenchyma does not generally form under well-drained soil conditions, but is induced by poor aeration (Arikado and Adachi, 1955; Drew *et al.*, 1981; Haque *et al.*, 2010).

Ethylene is involved in inducible aerenchyma formation (Jackson and Armstrong, 1999; Drew *et al.*, 2000; Evans, 2003). The treatment of maize roots with inhibitors of ethylene action or ethylene biosynthesis effectively blocks aerenchyma formation under hypoxic conditions (Drew *et al.*, 1981; Konings 1982; Jackson *et al.*, 1985*a*). Reactive oxygen species (ROS) are key factors that transduce signals stimulated by abiotic stresses in plants (Suzuki *et al.*, 2011). Respiratory burst oxidase homologue (RBOH), a plant homologue of gp91^phox^ in mammalian NADPH oxidase, has an important role in ROS-mediated signalling, such as the defence response, programmed cell death, and development in plants (Torres and Dangle, 2005). In rice (*Oryza sativa* L.), ethylene-induced hydrogen peroxide (H_2_O_2_)-mediated epidermal cell death during the emergence of adventitious roots is regulated by RBOH (Steffens and Sauter, 2009; Steffens *et al.*, 2012). On the other hand, application of H_2_O_2_ promotes lysigenous aerenchyma formation in internodes of rice stems (Steffens *et al.*, 2011). In cortical cells of maize roots, where lysigenous aerenchyma is developed, a gene encoding RBOH is strongly up-regulated under waterlogged conditions (Rajhi *et al.*, 2011), suggesting that the ROS generation mediated by RBOH at least partly contributes to the lysigenous aerenchyma formation in maize roots.

In this study, the effects of an ethylene precursor and an NADPH oxidase inhibitor on the tolerance of wheat seedlings to stagnant deoxygenated conditions (which mimic oxygen-deficient conditions in waterlogged soils), on the formation of lysigenous aerenchyma, and on the expressions of genes encoding ethanol fermentation enzymes in roots were examined. The results show that ethylene and ROS have important roles in each of these processes.

## Materials and methods

### Plant material and growth conditions

Spring wheat cv. Bobwhite line SH 98 26 was used for all experiments. Seeds were surface sterilized in 0.5% (v/v) sodium hypochlorite for 30min. These seeds were rinsed thoroughly with deionized water and subsequently germinated on moist filter paper in Petri dishes in a growth chamber at 23 °C under light conditions. After 1 d, germinated seeds were placed on a meshfloat with an aerated half-strength nutrient solution [23 °C, light conditions, photosynthetically active radiation (PAR), 200–250 μmol m^–2^ s^–1^] for 4 d. The composition of the nutrient solution was described by Colmer *et al.* (2006).

### Experimental design

To assess the effect of an ethylene precursor, 1-aminocyclopropanecarboxylic acid (ACC), on wheat adaptation to oxygen-deficient conditions, 5-day-old aerobically grown seedlings were transferred to 5 litre pots (9–12 plants per pot, 250mm height×120mm length×180mm width) containing an aerated full-strength nutrient solution with 20 μM ACC (Sigma-Aldrich, St. Louis, MO, USA). For a control, wheat seedlings were transferred to an aerated nutrient solution without ACC treatments. After 2 d, these seedlings were transferred to 5 litre pots containing an aerated full-strength nutrient solution (aerated conditions) or stagnant solution (stagnant conditions; Supplementary Fig. S1 available at *JXB* online). Stagnant solution contained 0.1% (w/v) dissolved agar and was deoxygenated (dissolved oxygen, <0.5mg l^–1^) prior to use by flushing with N_2_ gas. To assess the effect of an NADPH oxidase inhibitor, diphenyleneiodonium (DPI), on the ACC treatment-promoted tolerance to stagnant conditions, 5-day-old seedlings were transferred to aerated conditions with 0, 0.1, or 1 μM DPI (Sigma-Aldrich) together with 20 μM ACC (Supplementary Fig. S1).

### Growth measurements

Plants (14 d old) were harvested at 7 d after transfer to aerated conditions or stagnant conditions. The length and numbers of shoots, seminal roots, and adventitious roots were measured. Chlorophyll content of leaves was measured using a Soil Plant Analysis Development (SPAD) meter (SPAD-502, Konica Minolta, Tokyo, Japan). Chlorophyll meter readings were taken at the middle part of the leaves. Plants were divided into shoots and roots and dried for 7 d at 50 °C, and then shoot and root dry weights were measured. For the analysis of lateral root numbers and lengths, plants were harvested at 0h and 72h after transfer to stagnant conditions. Lateral root numbers were counted under a microscope (SZX16, OLYMPUS, Tokyo, Japan), and the lengths of lateral roots were measured with a ruler. Adventitious root numbers were counted each day during ACC pre-treatments and during subsequent treatment with aerated or stagnant conditions.

### Anatomical observations of roots

Root cross-sections were prepared from 4mm long root segments excised from seminal roots of wheat seedlings grown in either aerated or stagnant conditions with or without ACC and DPI pre-treatments. Root segments were prepared at 10, 30, 50, and 70mm from the root tips, and 10 and 30mm from the root–shoot junctions of the seminal roots. Cross-sections were prepared by hand-sectioning with a razor blade. Each section was photographed using an optical microscope (BX60, OLYMPUS) with a CCD camera (DP70, OLYMPUS). The percentage of each cross-section occupied by aerenchyma was determined using ImageJ software (Ver. 1.43u, US National Institutes of Health, Bethesda, MD, USA).

### TTC reduction assay

2,3,5-Triphenyltetrazolium chloride (TTC) is normally colourless, but turns red when reduced by dehydrogenases in living cells. TTC (Tokyo Chemical Industry Co., Ltd., Tokyo, Japan) was dissolved in 0.1M sodium phosphate buffer (pH 7.0) to a final concentration of 0.6% (w/v). Root segments prepared at distances from 0 to 30mm or from 30mm to 50mm from root tips of the first seminal roots were weighed (3.0–5.0mg) and transferred to 100 μl of TTC solution. After 30min incubation at 40 °C, TTC solution was removed and root segments were rinsed once with deionized water. For the extraction, the root segments were incubated overnight at room temperature in 150 μl of 95% (v/v) ethanol. The reduction of TTC was expressed as the absorbance of the extracted solutions at 520nm in a spectrophotometer (DU800, Beckman Coulter Inc., Brea, CA, USA). For TTC staining, root segments prepared at distances from 0 to 30mm from root tips of the first seminal roots were transferred to 500 μl of TTC solution and incubated at 40 °C for 30min. After staining, the root segments were photographed using an optical microscope (SZX16, OLYMPUS) with a CCD camera (DP71, OLYMPUS).

### RNA extraction and quantitative reverse transcription–PCR (qRT–PCR)

Total RNA was extracted from frozen fixed tissues from two sequential regions of the first seminal roots (i.e. 0–30mm and 30–50mm from the root tips, respectively) using an RNeasy Plant Mini Kit (Qiagen, Valencia, CA, USA) according to the manufacturer’s instructions. For qRT-PCR, 2ng of total RNA extracted from the first seminal roots were used as a template. Transcript levels were measured using a StepOnePlus Real-Time PCR System (Applied Biosystems, Foster City, CA, USA) and One Step SYBR PrimeScript RT-PCR Kit II (Takara Bio Inc., Otsu, Japan) in accordance with the manufacturers’ protocols. A PCR fragment of each gene was purified and quantified, and then was used to draw standard curves for absolute quantification. Subsequently, the quantified mRNA levels of each gene were normalized to mRNA levels of the *18S ribosomal RNA* gene as a control. qRT–PCR was performed using total RNA from three biological replicates. Primer sequences used for the experiments are shown in Supplementary Table S1 at *JXB* online. For *TaADH1* and *TaADH2* genes, two paralogues of *TaADH1* and three paralogues of *TaADH2* were archived in the database, and thus primers were designed to be able to amplify all of each paralogue.

### Ethylene measurement

Ethylene was measured by a modification of the method of Hattori *et al.* (2009). The aerial parts were excised from wheat seedlings, and the remaining underground parts were placed in a container with saturated NaCl solution. The gas in the container was deaerated with a vacuum pump, and the gas released from the wheat seedlings was collected in a test tube using a funnel. The collected gas was transferred to a gas chromatography vial, and the vial was fitted with a rubber stopper while held upside down in saturated NaCl solution. Then the vial was righted, an aliquot of the headspace gas in the vial was withdrawn with a syringe, and the ethylene content was measured by gas chromatography (GC 353, GL Sciences, Tokyo, Japan).

### Statistical analysis

Data are presented as the means ±SD. Statistical differences between means were calculated using two-sample *t*-test. For multiple comparisons, data were analysed by one-way analysis of variance (ANOVA) and post-hoc Tukey’s test using SPSS Statistics Version 19 (IBM Software, New York, NY, USA).

## Results

### Effect of ethylene on adaptation of wheat seedlings to oxygen-deficient conditions

**Fig. 1.**
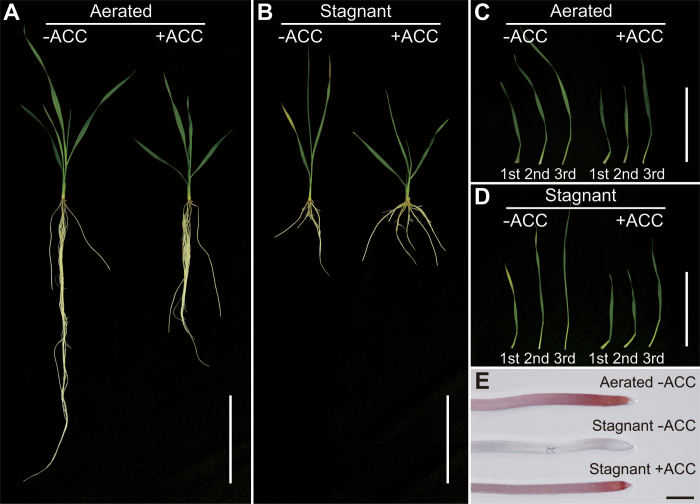
Wheat seedlings grown under aerated conditions (A) and stagnant conditions (B) for 7 d with or without 20 μM ACC pre-treatment. Leaves of wheat seedlings grown under aerated conditions (C) and stagnant conditions (D) for 7 d with or without 20 μM ACC pre-treatment. (E) First seminal root tips of wheat seedlings grown under aerated conditions for 3 d without 20 μM ACC pre-treatment and under stagnant conditions for 3 d with or without 20 μM ACC pre-treatment. Red color indicates TTC staining. Bars=100mm (A–D) and 1mm (E).

To assess the effect of ethylene on adaptation to oxygen-deficient conditions, 5-day-old wheat seedlings were pre-treated with an ethylene precursor, ACC (20 μM), under aerated conditions for 2 d. After 2 d of the ACC pre-treatment, reduction of the growth of shoots and first seminal roots was observed, while the numbers of leaves and the seminal roots were not affected (Supplementary Table S2 at *JXB* online). Subsequently, 7-day-old wheat seedlings were transferred to aerated or stagnant conditions. After 7 d (14 d old), growth of seedlings treated under each condition was measured (Fig. 1A, B; Table 1). Stagnant conditions reduced the shoot length of seedlings without the ACC pre-treatment (the ‘–ACC’ seedlings) by 25%, whereas the reduction was only by 8% in seedlings with the ACC pre-treatment (the ‘+ACC’ seedlings), compared with seedlings grown under aerated conditions (Table 1). The number of leaves was comparable (four leaves) between with and without ACC pre-treatment after 7 d growth under aerated conditions. Although stagnant conditions reduced the number of leaves to three in the ‘–ACC’ seedlings, the number of leaves (four leaves) was retained in the ‘+ACC’ seedlings (Table 1). Stagnant conditions reduced shoot dry weight of the ‘–ACC’ seedlings by 25%, but did not affect the dry weight of the ‘+ACC’ seedlings when compared with seedlings grown under aerated conditions (Table 1). The chlorophyll content (SPAD values) of leaves was significantly lower in the ‘–ACC’ seedlings grown under stagnant conditions when compared with seedlings grown under aerated conditions, while chlorophyll content was unaffected in the ‘+ACC’ seedlings (Fig. 1C, D; Table 1).

**Table 1.** Growth of wheat seedlings under aerated or stagnant conditions with or without ACC and DPI pre-treatments

Conditions	Before treatments (day 7)		After treatments (day 14)									
	Length (mm)		Length (mm)			Elongation (mm)		Number		Dry weight (mg)		SPAD
	Shoot	First seminal root	Shoot	First seminal root	Longest adventitious root	Shoot	First seminal root	Leaf	Adventitious root	Shoot	Root	First leaf
Aerated –ACC	120.2±4.49 a	118.6±6.91 a	240.0±6.22 a	361.8±10.89 a	126.8±7.56 a	119.8±7.03 a	243.2±11.34 a	4.0±0.00	2.6±0.73 a	110.4±8.50 a	66.1±6.79 a	44.4±2.32 a
Stagnant –ACC	115.9±2.62 a	117.3±4.30 a	180.8±13.62 b	ND	77.2±2.19 d	64.9±15.35 c	ND	3.0±0.00	4.9±0.78 b	83.3±5.70 b	27.8±3.35 d	21.3±6.78c
% of control^*a*^	96	99	75	–	61	54	–	75	191	75	42	48
Aerated +ACC	109.1±6.79 b	90.2±4.27 b	161.1±7.37 c	216.8±14.15 c	138.4±9.34 b	52.0±13.36 c,d	126.6±13.19 c	4.0±0.00	2.9±1.36 a	82.2±5.78 b	48.7±6.40 b	43.3±2.44 a
Stagnant +ACC	105.6±1.74 b	88.0±3.84 b	147.4±5.79 c	88.4±4.03 d	101.7±9.38 c	41.9±6.90 d	0.4±0.73 d	4.0±0.00	6.4±0.73 c	92.4±4.82 b	40.1±2.62 c	39.1±4.78 a,b
% of control^*a*^	97	98	92	41	73	81	0	100	223	112	82	90
Aerated +ACC +DPI	105.2±3.77 b	93.2±4.68 b	194.9±9.24 b	307.6±17.57 b	133.3±8.20 a,b	89.7±9.86 b	214.3±14.67 b	4.0±0.00	2.6±0.73 a	87.3±3.97 b.c	53.2±2.33 b	40.1±1.39 a,b
Stagnant +ACC +DPI	105.2±2.44 b	93.7±5.94 b	151.4±9.76 c	ND	91.8±3.50 d	46.2±10.18 d	ND	4.0±0.00	5.2±0.44 b	73.2±3.49 d	28.6±3.50 d	36.6±5.69 b
% of control^*a*^	100	101	78	–	69	52	–	100	204	84	54	91

Plants were grown in aerated or stagnant deoxygenated conditions for 7 d with or without 20 μM ACC and 0.1 μM DPI pre-treatment. Values are means (*n*=9) ±SD. Different lower case letters denote significant differences among each group parameter (*P* < 0.05, one-way ANOVA and then Tukey’s test for multiple comparisons). ND, not determined.

^*a*^ Relative ratio of parameters between aerated and stagnant conditions.

The lengths of the longest adventitious roots of the ‘–ACC’ seedlings and the ‘+ACC’ seedlings grown under stagnant conditions were reduced by 39% and 27%, respectively (Table 1). In contrast, the numbers of adventitious roots in the ‘–ACC’ seedlings and the ‘+ACC’ seedlings grown under stagnant conditions were increased by 91% and 123%, respectively (Table 1). Interestingly, in the first seminal roots of the ‘–ACC’ seedlings grown under stagnant conditions, the root tips died, whereas they did not die in the ‘+ACC’ seedlings. After 3 d, root tip cells were densely stained with TTC (indicating that the cells were alive) in the ‘+ACC’ seedlings grown under stagnant conditions, whereas little or no staining was observed at root tips of the ‘–ACC’ seedlings (Fig. 1E). Moreover, emergence and elongation of lateral roots were almost completely inhibited in the ‘–ACC’ seedlings grown under stagnant conditions (Supplementary Fig. S2 at *JXB* online). The root dry weights of the ‘–ACC’ and ‘+ACC’ seedlings under stagnant conditions were 58% less and 18% less, respectively, than the root dry weight of the seedlings grown under aerated conditions (Table 1).

### Induction of root aerenchyma formation by ACC pre-treatment in wheat seedlings

**Fig. 2.**
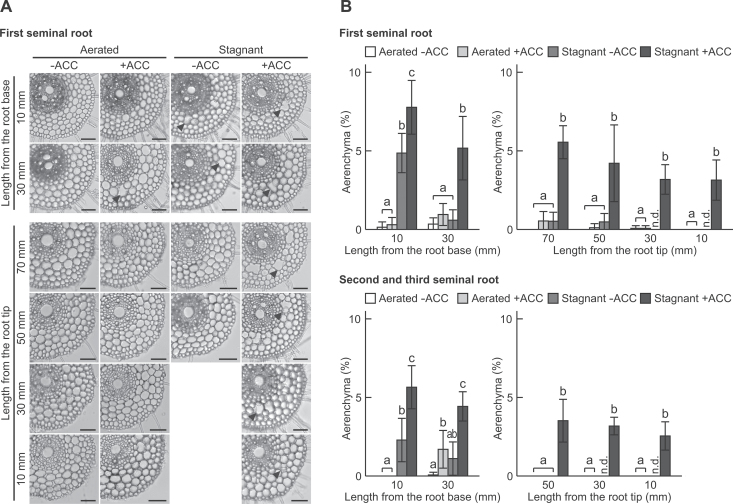
Formation of aerenchyma in seminal roots of wheat seedlings grown under aerated or stagnant conditions for 7 d with or without 20 μM ACC pre-treatment. (A) Cross-sections. Distances from the root tips and root–shoot junction (mm) are displayed on the left side of figures. Lysigenous aerenchyma is indicated by a black arrowhead. Bar=100 μm. (B) The percentage of aerenchyma of root cross-sectional area along first seminal roots, and second and third seminal roots of wheat seedlings grown under aerated or stagnant conditions for 7 d with or without 20 μM ACC pre-treatment. For the first seminal roots, the lengths were Aerated –ACC, 360–380mm; Aerated +ACC, 190–230mm; Stagnant –ACC, ~100–120mm; Stagnant +ACC, 80–100mm; and for the second and third seminal roots, the lengths were Aerated –ACC, 340–360mm; Aerated +ACC, 180–200mm; Stagnant –ACC, ~100–115mm; Stagnant +ACC, 80–90mm. Values are means (*n*=6) ±SD. Different lower case letters denote significant differences among the conditions (*P* < 0.05, one-way ANOVA and then Tukey’s test for multiple comparisons). n.d., not determined.

Aerenchyma formation in seminal roots of wheat seedlings grown under aerated or stagnant conditions for 7 d was measured. With ACC pre-treatment, aerenchyma formation was significantly increased in response to stagnant conditions, whereas aerenchyma formation hardly occurred under aerated conditions (Fig. 2). The percentage of the cross-section of the root occupied by aerenchyma in the ‘+ACC’ seedlings increased from 3.1% to 7.8% (Fig. 2B). The percentages of aerenchyma were comparable between the ‘–ACC’ seedlings grown under stagnant conditions and those grown under aerated conditions, except for the most basal position (Fig. 2B). Similar results were obtained in the second and third seminal roots (Fig. 2B).

### Time-course of aerenchyma formation and viability of first seminal roots with ACC pre-treatment

**Fig. 3.**
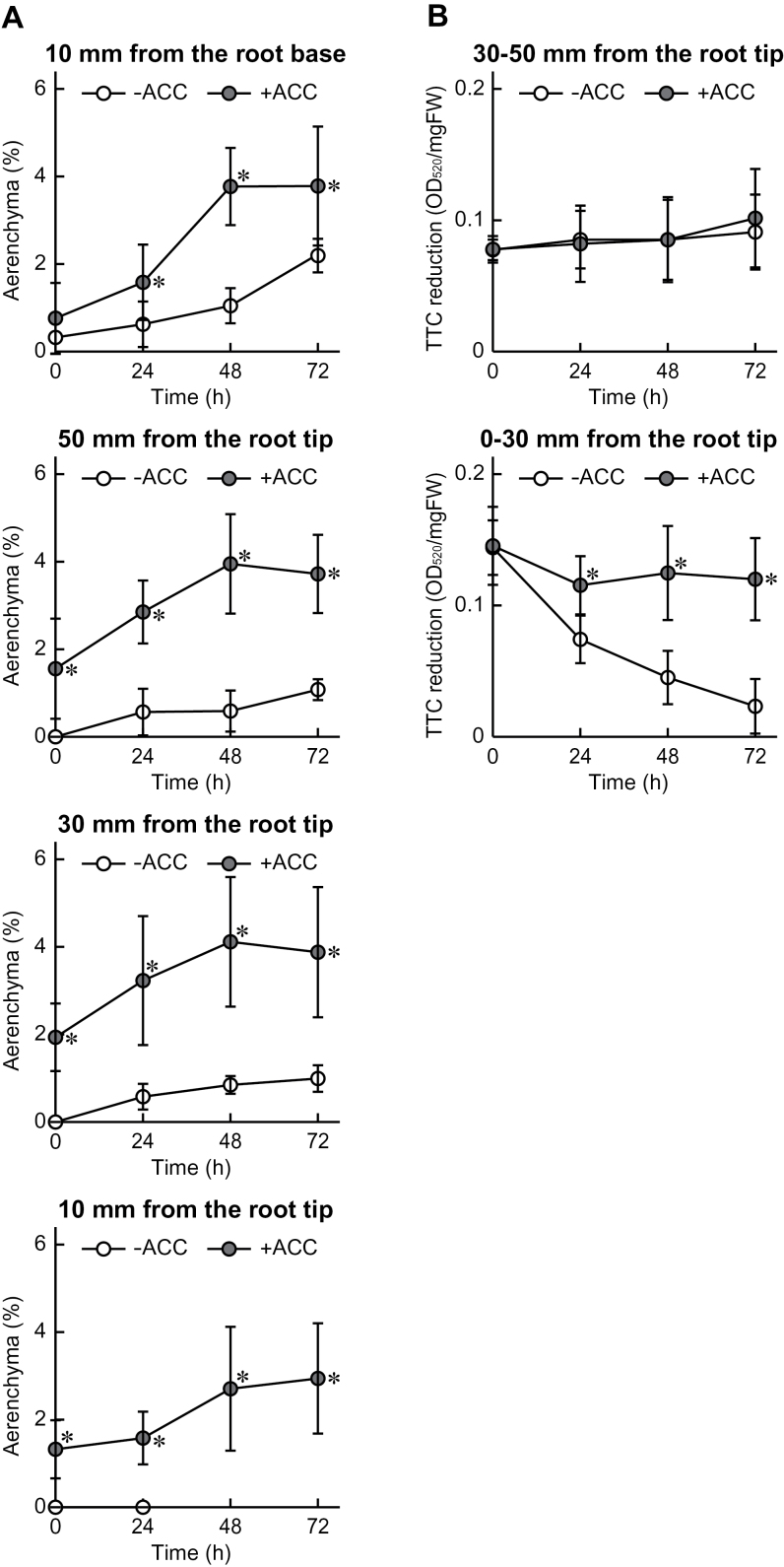
Time-course of aerenchyma formation in first seminal roots at 10mm from the root–shoot junction and at 10, 30, and 50mm from the root tips (A). Time-course of cell viability (TTC reduction) in first seminal roots at 0–30mm and 30–50mm from the root tips (B). Wheat seedlings were grown under stagnant conditions with or without 20 μM ACC pre-treatment. The lengths of the first seminal roots immediately after pre-treatment were 100–120mm without ACC pre-treatment and 80–100mm with ACC pre-treatment. Values are means (*n*=6) ±SD. A significant difference between with and without ACC pre-treatment at *P* < 0.05 (two-sample *t*-test) is denoted by *.

To understand further the relationship between aerenchyma formation and root tip death, the short-term time-courses of aerenchyma formation and TTC reduction were analysed in the first seminal roots of wheat seedlings treated with stagnant conditions with or without ACC pre-treatment. The lengths of the first seminal roots immediately after pre-treatments (0h) were 100–120mm without ACC pre-treatment and 80–100mm with ACC pre-treatment. As a result, aerenchyma formation in each position of the first seminal roots was enhanced during ACC pre-treatment (0h; Fig. 3A). The percentages of aerenchyma in the first seminal roots of the ‘+ACC’ seedlings gradually increased during growth under stagnant conditions and plateaued at 48h (Fig. 3A). The percentages of aerenchyma at 72h after growth under stagnant conditions reached 2.9, 3.9, and 3.7% at 10, 30, and 50mm from the root tips, respectively, and 3.8% at 10mm from the root–shoot junction (Fig. 3A). In contrast, aerenchyma formation hardly occurred at any of the positions of the first seminal roots of the ‘–ACC’ seedlings immediately after pre-treatment (0h; Fig. 3A). The percentages of aerenchyma increased to 1.0% and 1.1% at 30mm and 50mm from the root tips, respectively, and to 2.2% at 10mm from the root–shoot junction at 72h after growth under stagnant conditions (Fig. 3A). It should be noted that the cross-sections of the roots at 10mm from the root tips were hardly obtained because of the root tip death.

The TTC reduction assay has been used to evaluate viability of plant tissues quantitatively by detecting absorbance of reduced TTC, a red formazan derivative (Steponkus and Lanphear, 1967). At 0–30mm from the root tips, the values of TTC reduction (OD_520_) in the ‘–ACC’ seedlings were gradually reduced under stagnant conditions, whereas the value of TTC reduction in the ‘+ACC’ seedlings was kept higher (Fig. 3B). At 30–50mm from the root tips, no significant changes in the values of TTC reduction were observed between the ‘–ACC’ seedlings and the ‘+ACC’ seedlings (Fig. 3B).

The aerenchyma formation and TTC reduction analyses gave similar results when shorter first seminal roots (70–90mm) of 4-day-old wheat seedlings were used for ‘–ACC’ conditions (Supplementary Fig. S3 at *JXB* online).

### Expression of genes encoding ethanol fermentation enzymes and the PDH E1α subunit in first seminal roots

**Fig. 4.**
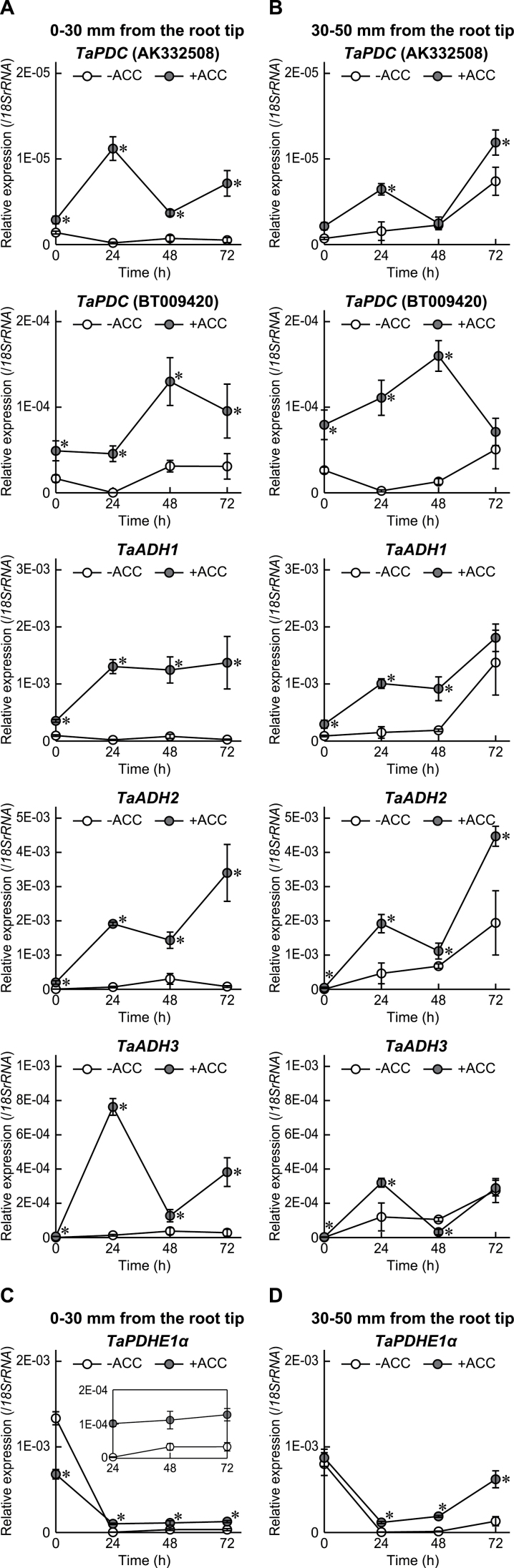
Time-course qRT–PCR analyses of *TaPDC* genes (AK332508 and BT009420), *TaADH1–3* genes (A and B), and the *TaPDHE1α* gene (C and D) were performed using RNAs extracted from first seminal roots at 0–30mm (A and C) and 30–50mm (B and D) from the root tips of wheat seedlings grown under stagnant conditions with or without 20 μM ACC pre-treatment. Values are means (*n*=3) ±SD. A significant difference between with and without ACC pre-treatment at *P* < 0.05 (two-sample *t*-test) is denoted by *.

To investigate whether the ACC pre-treatment induces expression of genes encoding ethanol fermentation enzymes in the first seminal roots, expression of two *PDC* genes, *TaPDC* genes, (accession nos AK332508 and BT009420), and three *ADH* genes, *TaADH1* (accession nos EF122847 and EF122848), *TaADH2* (accession nos EF122843, EF122844, and EF122845), and *TaADH3* (accession no. EF122842) (Supplementary Table S1 at *JXB* online), was analysed by qRT–PCR (Fig. 4A, B). The expression levels of the *TaPDC* genes and the *TaADH* genes at 0–30mm from the root tips in the first seminal roots immediately after ACC pre-treatment (0h) were significantly higher in the ‘+ACC’ seedlings than in the ‘–ACC’ seedlings, and thereafter their expression was further increased in the ‘+ACC’ seedlings (Fig. 4A). It should be noted that the lower expression levels of the genes at 0–30mm from the root tips in the first seminal roots of the ‘–ACC’ seedlings grown under stagnant conditions could be largely due to the lower cell viability (Fig. 3B). At 30–50mm from the root tips, the expression levels of the *TaPDC* (BT009420) gene, and the *TaADH1*, *2*, and *3* genes immediately after pre-treatment (0h) were significantly higher in the ‘+ACC’ seedlings than in the ‘–ACC’ seedlings (Fig. 4B). Expression of all genes encoding ethanol fermentation enzymes in the ‘+ACC’ seedlings and the ‘–ACC’ seedlings were induced under stagnant conditions, but their expression levels were higher in the ‘+ACC’ seedlings than in the ‘–ACC’ seedlings (Fig. 4B). The expression level of the *TaPDC* (BT009420) gene was higher than that of the *TaPDC* (AK332508) gene (Fig. 4), and the expression level of the *TaADH1* gene was highest among the three *TaADH* genes immediately after ACC pre-treatment (0h), whereas *TaADH2* gene expression was highest at 72h after initiation of the treatments (Fig. 4).

The mitochondrial pyruvate dehydrogenase (PDH) complex plays a pivotal role in the tricarboxylic acid (TCA) cycle and following oxidative phosphorylation by the conversion of pyruvate to acetyl-CoA in aerobic cells (Patel and Roche, 1990). The activity of the mitochondrial PDH complex depends on the expression of the gene encoding PDH E1α (Luethy *et al.*, 2001), which is one of two subunits of the mitochondrial PDH E1 component (Mooney *et al.*, 2002). To assess whether the induction of aerenchyma formation in the first seminal roots of wheat seedlings affects the expression level of the wheat *PDH E1α* (*TaPDHE1α*) gene, qRT–PCR analysis of the *TaPDHE1α* gene (accession no. GU563379) (Supplementary Table S1 at *JXB* online) was performed (Fig. 4C, D). Although the expression level of the *TaPDHE1α* gene was reduced under stagnant conditions, reduction of the expression in the first seminal roots was significantly lower in the ‘+ACC’ seedlings than in the ‘–ACC’ seedlings (Fig. 4C, D). Interestingly, the expression level of *TaPDHE1α* at 30–50mm from the root tips of the first seminal roots of the ‘+ACC’ seedlings started to increase at 48h after initiation of growth under stagnant conditions (Fig. 4D), implying that the internal transport of oxygen from shoot to roots by forming the aerenchyma leads to the recovery of *TaPDHE1α* expression.

### Ethylene accumulation in roots and expression of one of genes encoding ACC synthase (ACS) in first seminal roots

**Fig. 5.**
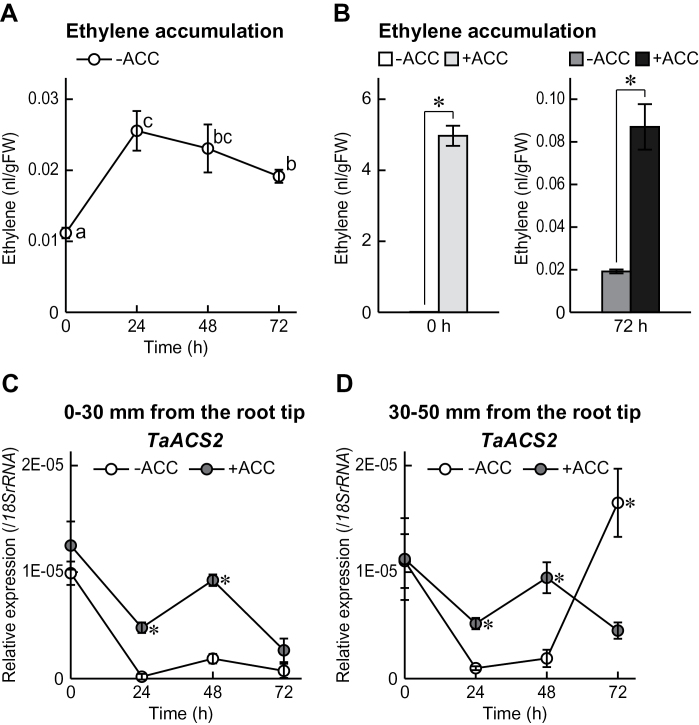
Time course of ethylene contents in roots of wheat seedlings grown under aerated or stagnant conditions without ACC pre-treatment (A). Ethylene was measured in roots of wheat seedlings at 0h and 72h after initiation of growth under stagnant conditions with or without 20 μM ACC pre-treatment (B). Time-course qRT–PCR analysis of the *TaACS2* gene was performed using RNAs extracted from first seminal roots at 0–30mm (C) and 30–50mm (D) from the root tips of wheat seedlings grown under stagnant conditions with or without 20 μM ACC pre-treatment. Values are means (*n*=3) ±SD. Different lower case letters denote significant difference among the conditions (*P* < 0.05, one-way ANOVA and then Tukey’s test for multiple comparisons). A significant difference between with and without ACC pre-treatment at *P* < 0.05 (two-sample *t*-test) is denoted by *.

To assess whether ethylene accumulated in roots of wheat seedlings grown under stagnant conditions with or without ACC pre-treatments, ethylene was measured in roots and the expression of a gene encoding ACS (*TaACS2*; accession no. U42336, a key enzyme of ethylene biosynthesis) was measured in first seminal roots of wheat seedlings (Fig. 5). The content of ethylene in the roots of the ‘–ACC’ seedlings increased ~2.3-fold at 24h after initiation of growth under stagnant conditions, and then gradually decreased (Fig. 5A). The ethylene contents were ~500 times higher in the roots of the ‘+ACC’ seedlings than in those of the ‘–ACC’ seedlings immediately after pre-treatments (0h; Fig. 5B). The ethylene contents decreased in the roots of the ‘+ACC’ seedlings at 72h after initiation of growth under stagnant conditions, possibly because ACC was not included in the stagnant solution. However, the ethylene contents were still ~5 times higher in the ‘+ACC’ seedlings than in the ‘–ACC’ seedlings (Fig. 5B).

The *TaACS2* gene is predominantly expressed in roots of wheat seedlings (Subramaniam *et al.*, 1996). The *TaACS2* gene expression was measured at two locations in the first seminal roots of wheat seedlings after transfer to stagnant conditions. At 0–30mm from the root tips, the *TaACS2* expression immediately after ACC pre-treatment (0h) was comparable between the ‘+ACC’ and the ‘–ACC’ seedlings, but was significantly higher in the ‘+ACC’ seedlings at 24h and 48h (Fig. 5C). At 30–50mm from the root tips, the *TaACS2* expression was significantly higher in the ‘+ACC’ seedlings at 24h and 48h, but was much higher in the ‘–ACC’ seedlings at 72h (Fig. 5D).

### Expression of genes encoding RBOH in the first seminal roots

**Fig. 6.**
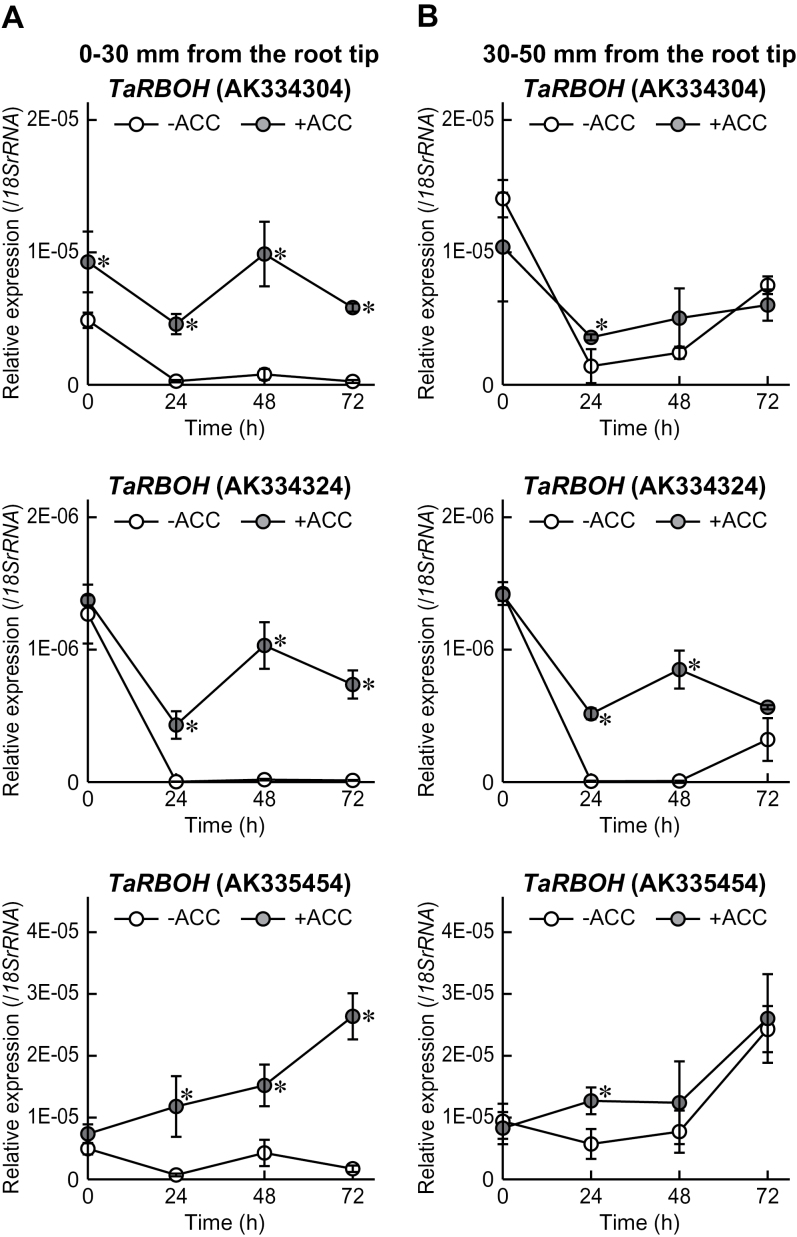
Time-course qRT–PCR analyses of *TaRBOH* genes (AK334304, AK334324, and AK335454) were performed using RNAs extracted from first seminal roots at 0–30mm (A) and 30–50mm (B) from the root tips of wheat seedlings grown under stagnant conditions with or without 20 μM ACC pre-treatment. Values are means (*n*=3) ±SD. A significant difference between with and without ACC pre-treatment at *P* < 0.05 (two-sample *t*-test) is denoted by *.

To investigate expression levels of *RBOH* genes in first seminal roots of wheat seedlings, three *TaRBOH* genes (accession nos AK334304, AK334324, and AK335454) were selected (Supplementary Table S1 at *JXB* online). Expression levels of the *TaRBOH* (AK334304) and *TaRBOH* (AK334324) genes at 0–30mm and 30–50mm from the root tips were decreased in the ‘–ACC’ seedlings under stagnant conditions, whereas the levels were kept higher in the ‘+ACC’ seedlings than in the ‘–ACC’ seedlings (Fig. 6). On the other hand, the expression levels of the *TaRBOH* (AK335454) gene at 0–30mm from the root tips was gradually increased during growth of the ‘+ACC’ seedlings, but not of the ‘–ACC’ seedlings, under stagnant conditions (Fig. 6A). At 30–50mm from the root tips, *TaRBOH* (AK335454) expression was increased in both the ‘+ACC’ seedlings and the ‘–ACC’ seedlings under stagnant conditions, but its levels were slightly higher in the ‘+ACC’ seedlings than in the ‘–ACC’ seedlings (Fig. 6B). Among three *TaRBOH* genes, the expression level of the *TaRBOH* (AK334304) gene was highest in the first seminal roots immediately after ACC pre-treatment (0h), whereas *TaRBOH* (AK335454) expression was highest at 72h after initiation of growth under stagnant conditions (Fig. 6).

### Aerenchyma formation and viability of first seminal roots with DPI pre-treatment

**Fig. 7.**
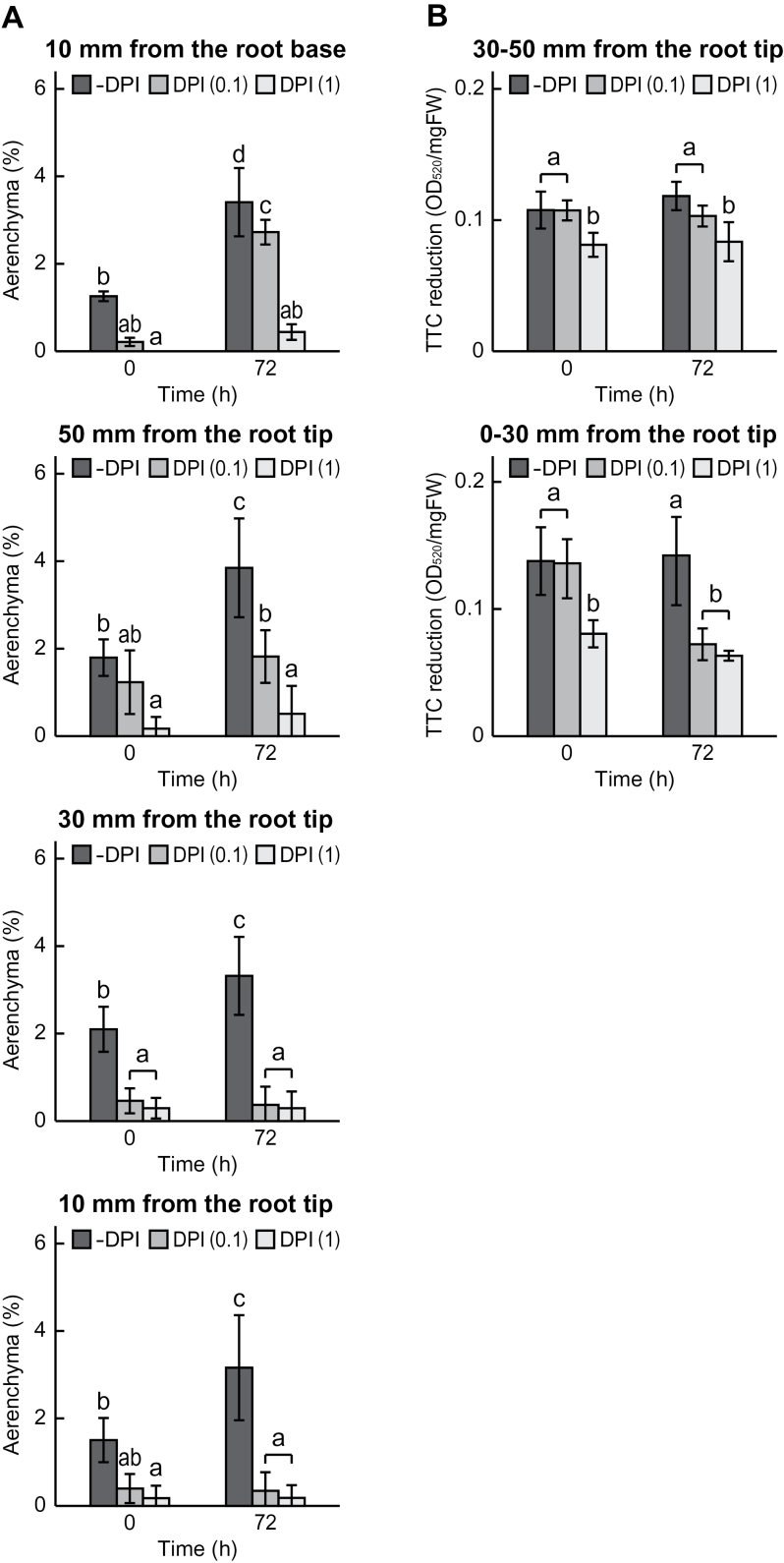
Aerenchyma formation in first seminal roots at 10mm from the root–shoot junction and at 10, 30, and 50mm from the root tips (A). Cell viability (TTC reduction) of first seminal roots at 0–30mm and 30–50mm from the root tips (B). Wheat seedlings were grown under stagnant conditions with pre-treatment with different concentrations of DPI (0, 0.1, and 1 μM) together with 20 μM ACC. The lengths of the first seminal roots immediately after 0, 0.1, and 1 μM DPI pre-treatment were 80–100, 80–100, and 60–80mm, respectively. Values are means (*n*=6) ±SD. Different lower case letters denote significant differences among the conditions and among the time points (*P* < 0.05, one-way ANOVA and then Tukey’s test for multiple comparisons).

To understand the effect of an NADPH oxidase inhibitor, DPI, on lysigenous aerenchyma formation in first seminal roots of wheat seedlings, the percentage of aerenchyma was investigated and compared among first seminal roots of seedlings treated under stagnant conditions with 2 d pre-treatment with different concentrations of DPI (0, 0.1, and 1 μM) together with 20 μM ACC. The lengths of the first seminal roots immediately after 0, 0.1, and 1 μM DPI pre-treatment (0h) were 80–100, 80–100, and 60–80mm, respectively. The ACC-induced aerenchyma formation at each position of the first seminal roots at 0h was significantly reduced by the DPI pre-treatment in a dose-dependent manner (Fig. 7A). At 72h after initiation of growth under stagnant conditions, the ACC-induced aerenchyma formation was also significantly reduced by the DPI pre-treatment in a dose-dependent manner (Fig. 7A).

TTC reduction assay was performed using segments at 0–30mm and 30–50mm from the root tips. The value of TTC reduction of the first seminal roots was not affected by 0.1 μM DPI pre-treatment, but was reduced by 1 μM DPI pre-treatment (0h) (Fig. 7B). At 0–30mm from the root tips of the first seminal roots with 0.1 μM DPI pre-treatment, the value of TTC reduction was significantly reduced at 72h after initiation of growth under stagnant conditions, while the values without DPI pre-treatment was relatively the same between at 0h and 72h (Fig. 7B). At 30–50mm from the root tips, no significant changes in the values of TTC reduction were observed between at 0h and 72h (Fig. 7B).

### Expression of the genes in first seminal roots with DPI pre-treatment

**Fig. 8.**
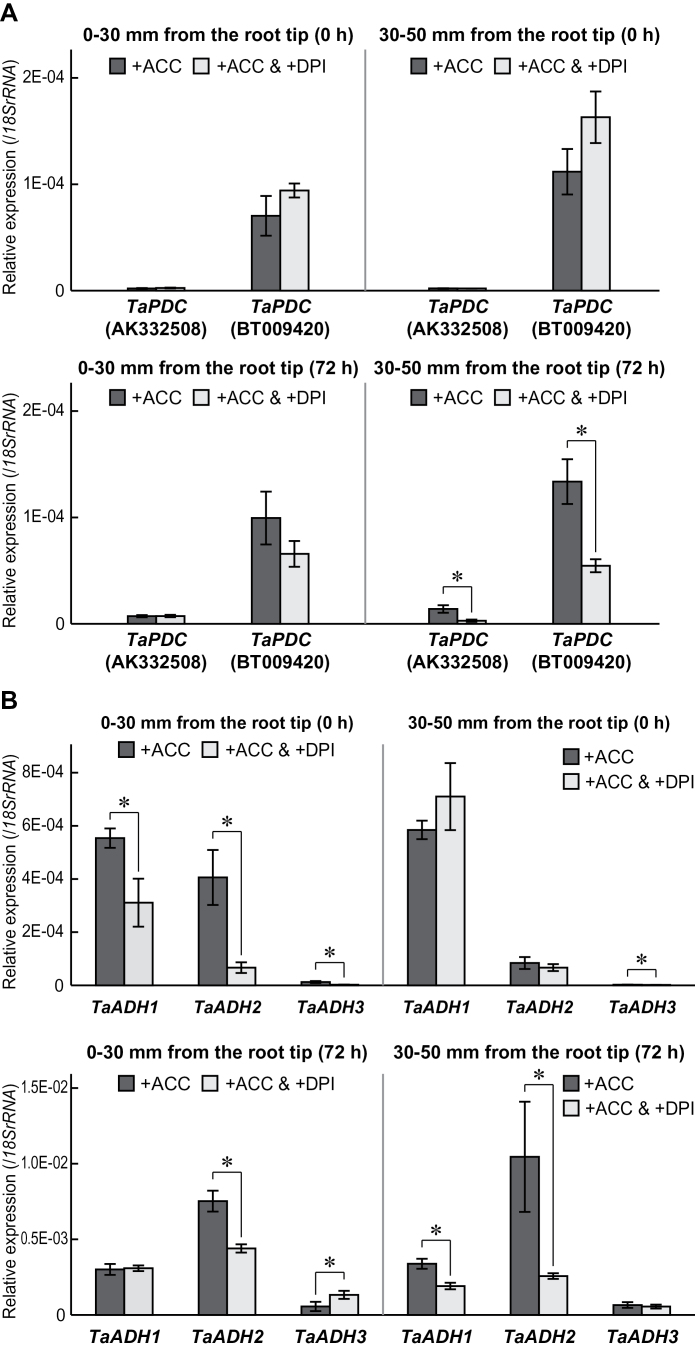
qRT–PCR analyses of *TaPDC* genes (AK332508 and BT009420) (A) and *TaADH1–3* genes (B) were performed using RNAs extracted from first seminal roots at 0–30mm and 30–50mm from the root tips of wheat seedlings grown under stagnant conditions with or without pre-treatment with 0.1 μM DPI together with 20 μM ACC. Values are means (*n*=3) ±SD. A significant difference between with and without DPI pre-treatment at *P* < 0.05 (two-sample *t*-test) is denoted by *.

qRT–PCR was performed to investigate whether the DPI pre-treatment represses ACC-induced expression of the *TaADH* and the *TaPDC* genes in the first seminal roots of wheat seedlings. During the pre-treatment, the expression of the *TaPDC* (AK332508) and the *TaPDC* (BT009420) genes in first seminal roots of seedlings were comparable between pre-treatment with 20 μM ACC (the ‘+ACC’ seedlings) and pre-treatment with 20 μM ACC and 0.1 μM DPI (the ‘+ACC & +DPI’ seedlings) (Fig. 8A). However, the expression levels of the *TaADH1*, *2*, and *3* genes were significantly reduced at 0–30mm from the root tips of the first seminal roots of the ‘+ACC & +DPI’ seedlings, compared with the ‘+ACC’ seedlings (Fig. 8B). At 30–50mm from the root tips, the *TaADH3* gene expression was significantly reduced in the first seminal roots of the ‘+ACC & +DPI’ seedlings (0h; Fig. 8B). At 72h, significant reductions in *TaPDC* gene expression were observed at 30–50mm from the root tips (Fig. 8A). The expression of the *TaADH2* gene was significantly reduced at 0–30mm from the root tips of the first seminal roots of the ‘+ACC & +DPI’ seedlings (Fig. 8B). At 30–50mm from the root tips, the expression of the *TaADH1* and the *TaADH2* genes was reduced in the first seminal roots of the ‘+ACC & +DPI’ seedlings (Fig. 8B). Although the expression level of the *TaPDHE1α* gene was reduced under stagnant conditions, reduction of the expression in the first seminal roots was significantly higher in the ‘+ACC & +DPI’ seedlings than in the ‘+ACC’ seedlings (Supplementary Fig. S4A, B at *JXB* online). The expression level of the *TaACS2* gene was comparable between the ‘+ACC’ seedlings and the ‘+ACC & +DPI’ seedlings at 0h and 72h (Supplementary Fig. S4C, D), suggesting that ethylene contents in the roots of the ‘+ACC & +DPI’ seedlings were identical to those of the ‘+ACC’ seedlings. The *RBOH* expression is considered a reliable indicator of ROS production because treatment with H_2_O_2_ induced transcription of the *RBOH* gene in *Arabidopsis* (Desikan *et al.* 1998) and maize (Lin *et al.* 2009), and treatment with scavengers of H_2_O_2_ suppressed induction of *RBOH* expression in maize (Lin *et al.*, 2009). The expression levels of the *TaRBOH* genes in the first seminal roots of the ‘+ACC & +DPI’ seedlings were significantly lower than those in the ‘+ACC’ seedlings (Supplementary Fig. S5), supporting that ROS production was severely depressed by DPI pre-treatment.

### Effect of the DPI treatment on wheat adaptation to oxygen-deficient conditions

To assess the effect of the DPI treatment on the adaptation to oxygen-deficient conditions in wheat seedlings, 5-day-old seedlings were pre-treated with 0.1 μM DPI together with 20 μM ACC for 2 d in aerated conditions. Subsequently, 7-day-old wheat seedlings were transferred to aerated or stagnant conditions (Supplementary Fig. S1 at *JXB* online). After 7 d (14 d old), growth of seedlings treated with each condition was measured (Table 1; Supplementary Fig. S6 at *JXB* online). Stagnant conditions reduced the shoot length of the ‘+ACC & +DPI’ seedlings by 22%, and decreased shoot dry weight by 16%, compared with the seedlings grown under aerated conditions (Table 1). Leaf number was reduced to three in the ‘+ACC & +DPI’ seedlings grown under stagnant conditions (Table 1). The longest adventitious root length and root dry weight in stagnant conditions were reduced by 31% and 46%, respectively, for the ‘+ACC & +DPI’ seedlings, compared with seedlings grown under aerated conditions (Table 1).

## Discussion

### Ethylene and ROS signalling are involved in wheat adaptation to oxygen-deficient conditions

Stagnant conditions severely reduced shoot length, leaf number, and shoot and root dry weights of wheat seedlings (Fig. 1A, B; Table 1), whereas all of the reductions of these growth parameters were alleviated by pre-treatment with the ethylene precursor ACC (Fig. 1A, B; Table 1). Exposure of several plants to hypoxic conditions greatly improved their anoxic stress tolerance (Waters *et al.*, 1991; Dolferus *et al.*, 1997; Germain *et al.*, 1997; Ellis and Setter, 1999). Because the effects of ACC are similar to the effects of hypoxic pre-treatments in several plants, ethylene may be a factor involved in the induction of the adaptive responses during hypoxic pre-treatments. Hormonal signalling pathways regulated by ethylene and ROS are involved in the adaptation of plants to abiotic stress (Overmyer *et al.*, 2003; Fujita *et al.*, 2006; Mittler *et al.*, 2011). The cancelling of the effect of ACC by DPI (Table 1; Supplementary Fig. S6 at *JXB* online) suggests that ethylene-mediated ROS signalling plays a role in the adaptation of wheat seedlings to oxygen-deficient conditions. Leaf chlorosis has been used to evaluate the tolerance of plants to waterlogging (Zhou, 2011; Mano and Takeda, 2012) because nutrient uptake and photosynthesis are severely affected by waterlogging (Trought and Drew, 1980*b*, *c*). The finding that ACC prevented the decrease of chlorophyll content of leaves grown under stagnant conditions (Fig. 1C, D; Table 1) is another indication that ACC enhanced the tolerance of wheat seedlings to oxygen-deficient conditions.

### Expression of genes encoding ethanol fermentation enzymes in first seminal roots of wheat seedlings was induced by ethylene and the ROS signalling pathway

The ACC pre-treatment significantly induced the expression levels of the *TaPDC* genes and the *TaADH* genes in the first seminal roots (Fig. 4), indicating that the induction of expression of the genes encoding ethanol fermentation enzymes is regulated by ethylene. Subsequent growth under stagnant conditions further induced the expression levels of the *TaPDC* genes and the *TaADH* genes, suggesting that ethylene itself was not sufficient for full induction of expressions of these genes. This result is supported by the study in *Arabidopsis* showing that the hypoxic induction of the *ADH* gene could not be completely suppressed by the treatment with an inhibitor of ethylene biosynthesis (Peng *et al.*, 2001). Production of H_2_O_2_ via a DPI-sensitive NADPH oxidase is necessary for the induction of *ADH* gene expression as well as the enhancement of the ADH activity in *Arabidopsis* (Baxter-Burrell *et al.*, 2002). Because the expression level of three homologues of NADPH oxidase in wheat was significantly higher in the first seminal roots of the ‘+ACC’ seedlings than in those of the ‘–ACC’ seedlings (Fig. 6A), RBOH-mediated ROS generation may be stimulated by ethylene in the first seminal roots. The co-treatment of ACC and DPI partly suppressed the ACC-induced expression of the *TaADH* genes in the first seminal roots at 0–30mm from the root tips (0h; Fig. 8B), suggesting that expression of the *TaADH* genes is regulated not only by ethylene but also by ROS. At 72h after initiation of growth under stagnant conditions, the expression of the *TaADH2* gene was also significantly reduced in the first seminal roots at 0–30mm from the root tips of the ‘+ACC & +DPI’ seedlings when compared with the ‘+ACC’ seedlings (Fig. 8B). Since the *TaADH2* gene expression was highest at 72h after initiation of the treatments (Fig. 8B), the protein levels and the activity of ADH in roots of the wheat seedlings may be stimulated by ethylene-mediated ROS signalling. The expression of the *TaADH1* and *TaADH2* genes and of the *TaPDC* genes in the first seminal roots at 30–50mm from the root tips was significantly reduced by the DPI pre-treatment at 72h after initiation of growth under stagnant conditions (Fig. 8). These results suggest that the expression levels of the genes encoding ethanol fermentation enzymes were regulated by both ethylene and ROS signalling.

### Induction of lysigenous aerenchyma formation in first seminal roots of wheat seedlings was mediated by ethylene and the ROS signalling pathway

Aerenchyma formation in each position of the first seminal roots of wheat seedlings was enhanced by ACC pre-treatment (Fig. 3A), and ACC-induced aerenchyma formation was significantly inhibited by the DPI pre-treatment in a dose-dependent manner (Fig. 7A). During the aerenchyma formation in maize roots, expression of a gene encoding RBOH is induced in the cortical cells of the primary roots, and its induction is suppressed by pre-treatment with an inhibitor of ethylene perception (Rajhi *et al.*, 2011). Moreover, aerenchyma formation of maize roots under waterlogged conditions was inhibited by the treatment with DPI (Yamauchi *et al.*, 2011). Together, ethylene-induced RBOH-mediated ROS generation may be commonly involved in lysigenous aerenchyma formation in roots of cereal crops (Nishiuchi *et al.*, 2012; Yamauchi *et al.*, 2013).

### Ethylene accumulation in roots is essential for the adaptive responses of wheat seedlings to oxygen-deficient conditions

Although the accumulation of ethylene in the roots of the ‘–ACC’ seedlings peaked at 24h after initiation of growth under stagnant conditions (Fig. 5A), the *TaACS2* expression in the first seminal roots of the ‘–ACC’ seedlings dropped to a low level at 24h and 48h after initiation of growth under stagnant conditions (Fig. 5C, D). These observations imply that the initial accumulation of ethylene mainly occurred through ‘physical entrapment’ of ethylene gas as a result of the low rate of gas diffusion under stagnant conditions. Under aerated conditions, adventitious roots of the ‘–ACC’ seedlings first emerged at 72h after the initiation of growth, while under stagnant conditions they first emerged at 48h (Supplementary Fig. S7 at *JXB* online). Therefore, initially, ethylene accumulated predominantly at the most basal part of the roots where adventitious roots emerged, raising the possibility that it contributes to the formation of the adventitious roots. In rice, adventitious root emergence under submergence is thought to be stimulated by ethylene accumulation through both increased ethylene biosynthesis and physical entrapment (Lorbiecke and Sauter, 1999; Sauter, 2013). In spite of the early induction of the ethylene accumulation in the roots of the ‘–ACC’ seedlings, the expression level of the *TaACS2* gene was strongly induced at 72h after initiation of growth under stagnant conditions (Fig. 5C, D). The aerenchyma formation and the expression levels of the genes encoding ADH, PDC, and RBOH at 30–50mm from the root tips in the first seminal roots of the ‘–ACC’ seedlings were also increased at 72h after initiation of growth under stagnant conditions (Figs 3A, 4B, 6B). These results suggest that local accumulation of ethylene stimulated by ethylene biosynthesis is essential for the adaptive responses to oxygen-deficient conditions. If this is the case, the late induction of *TaACS2* gene expression may be one of the reasons why wheat is less tolerant to oxygen-deficient conditions in waterlogged soils than wetland species. ACC pre-treatment remarkably enhanced the accumulation of ethylene in the roots of wheat seedlings even under the aerated conditions (at 0h; Fig. 5B), indicating that the roots of the ‘+ACC’ seedlings were ready to respond to oxygen-deficient conditions.

### Lysigenous aerenchyma has a role in sustaining aerobic respiration through the internal transport of oxygen into roots of wheat seedlings

Stagnant conditions severely reduced the expression level of the *TaPDHE1α* gene, but the reduction of the expression level was alleviated by the ACC pre-treatment in the first seminal roots (Fig. 4C, D). Interestingly, the expression level of the *TaPDHE1α* gene at 30–50mm from the root tips of the first seminal roots of the ‘+ACC’ seedlings started to increase at 48h after initiation of growth under stagnant conditions. The percentage aerenchyma formation plateaued at 48h after initiation of growth under stagnant conditions, supporting that the internal transport of oxygen to roots through the aerenchyma contributes to sustaining the level of aerobic respiration through the enhancement of *TaPDHE1α* expression. Oxygen is internally transported to roots through the aerenchyma (Armstrong, 1979; Colmer, 2003). Moreover, aerenchymatous roots of maize showed higher values for ATP content, adenylate energy charge, and ATP/ADP ratios than non-aerenchymatous roots in anaerobic conditions (Drew *et al.*, 1985). A relatively high level of aerobic respiration can be sustained for the roots with extensively formed aerenchyma, even in anoxic conditions. In the present study, it was also demonstrated that both the aerenchyma formation and the expression level of the *TaPDHE1α* gene under stagnant conditions were significantly reduced in the first seminal roots of the ‘+ACC & +DPI’ seedlings when compared with the ‘+ACC’ seedlings (Fig. 7A; Supplementary Fig. S4A, B at *JXB* online). These results further support the correlation between aerobic respiration and aerenchyma formation.

In conclusion, it was found that the formation of lysigenous aerenchyma and the expression levels of the genes encoding ethanol fermentation enzymes were enhanced in the first seminal roots of the ACC-pre-treated seedlings that were further grown under stagnant conditions. Moreover, DPI pre-treatment mostly diminished the effect of ACC on the adaptive response to stagnant conditions. These results suggest that ethylene-mediated ROS signalling is involved in regulating the adaptive responses to oxygen-deficient conditions in waterlogged soil.

## Supplementary data

Supplementary data are available at *JXB* online.

Figure S1. Growth conditions to assess the effect of the ethylene precursor, ACC, and the NADPH oxidase inhibitor, DPI, on adaptation of wheat seedlings to oxygen-deficient conditions.

Figure S2. Lateral root numbers and longest lateral root lengths of wheat seedlings in stagnant conditions with or without ACC pre-treatment.

Figure S3. Time-course of aerenchyma formation and cell viability in shorter first seminal roots of wheat seedlings in stagnant conditions with or without ACC pre-treatment.

Figure S4. qRT–PCR analyses of *TaPDHE1α* and *TaACS2* genes in first seminal roots of wheat seedlings in stagnant conditions with or without pre-treatment with DPI together with ACC.

Figure S5. qRT–PCR analyses of *TaRBOH* genes (AK334304, AK334324, and AK335454) in first seminal roots of wheat seedlings in stagnant conditions with or without pre-treatment with DPI together with ACC.

Figure S6. Growth of wheat seedlings under aerated conditions and stagnant conditions with or without pre-treatment with DPI together with ACC.

Figure S7. Time-course of emerged adventitious root numbers of wheat seedlings in aerated or stagnant conditions with or without ACC pre-treatment.

Supplementary Data
