# Supplementary material for: Ethylene and reactive oxygen species are involved in root aerenchyma formation and adaptation of wheat seedlings to oxygen-deficient conditions
Source: J Exp Bot. 2013 Nov 19;65(1):261–73. doi: 10.1093/jxb/ert371 (PMC3883296; doi:10.1093/jxb/ert371)
Supplement: Supplementary Data [file supp_65_1_261__index.html]

Ethylene and reactive oxygen species are involved in root aerenchyma formation and adaptation of wheat seedlings to oxygen-deficient conditions — Ethylene and reactive oxygen species are involved in root aerenchyma formation and adaptation of wheat seedlings to oxygen-deficient conditions — Supplementary Data 

# Ethylene and reactive oxygen species are involved in root aerenchyma formation and adaptation of wheat seedlings to oxygen-deficient conditions

## Supplementary Data

Data files

**Files in this Data Supplement:**

- Supplementary Data - Supplementary Data
