# Supplementary material for: Ethylene and reactive oxygen species are involved in root aerenchyma formation and adaptation of wheat seedlings to oxygen-deficient conditions
Source: J Exp Bot. 2013 Nov 19;65(1):261–73. doi: 10.1093/jxb/ert371 (PMC3883296; doi:10.1093/jxb/ert371)
Supplement: Supplementary Data [file supp_ert371_jexbot105692_file001.pdf]

Supplementary Material

Ethylene and reactive oxygen species are involved in root aerenchyma formation and adaptation of wheat seedlings to oxygen-deficient conditions

Takaki Yamauchi, Kohtaro Watanabe, Aya Fukazawa, Hitoshi Mori, Fumitaka Abe, Kentaro Kawaguchi, Atsushi Oyanagi and Mikio Nakazono

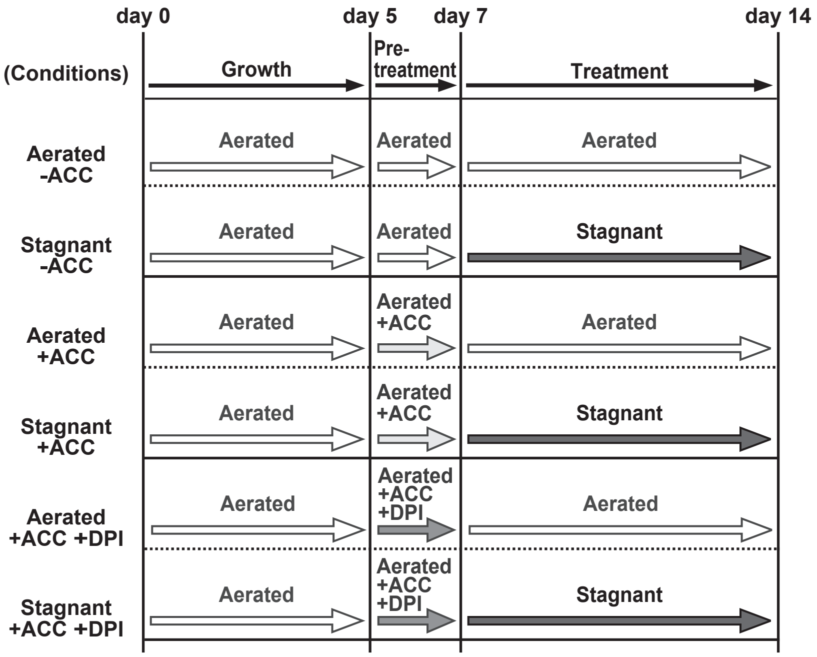

Supplementary Figure S1. Yamauchi et al.

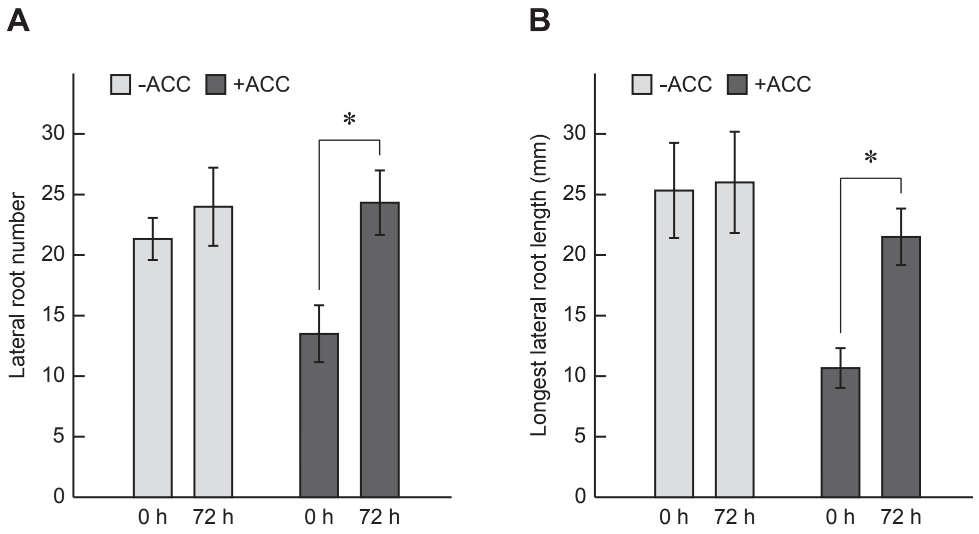

Supplementary Figure S2. Yamauchi et al.

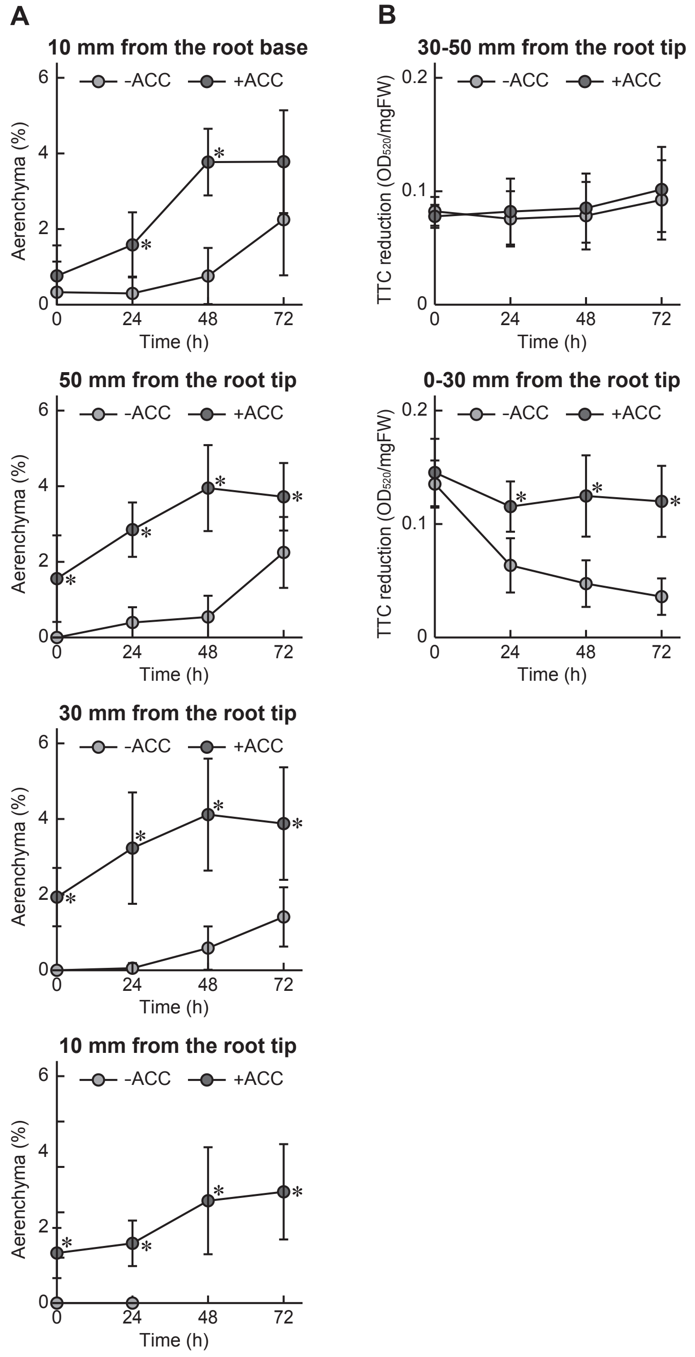

Supplementary Figure S3. Yamauchi et al.

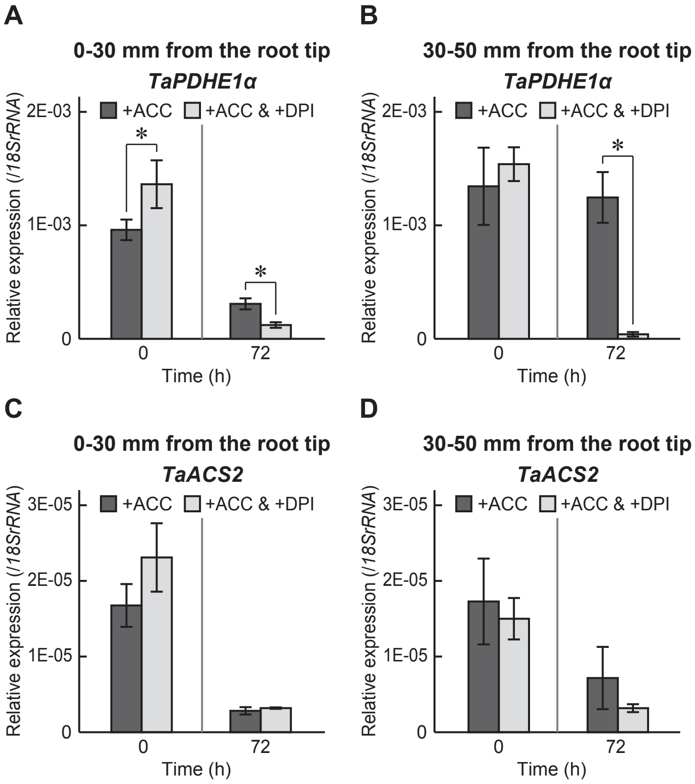

Supplementary Figure S4. Yamauchi et al.

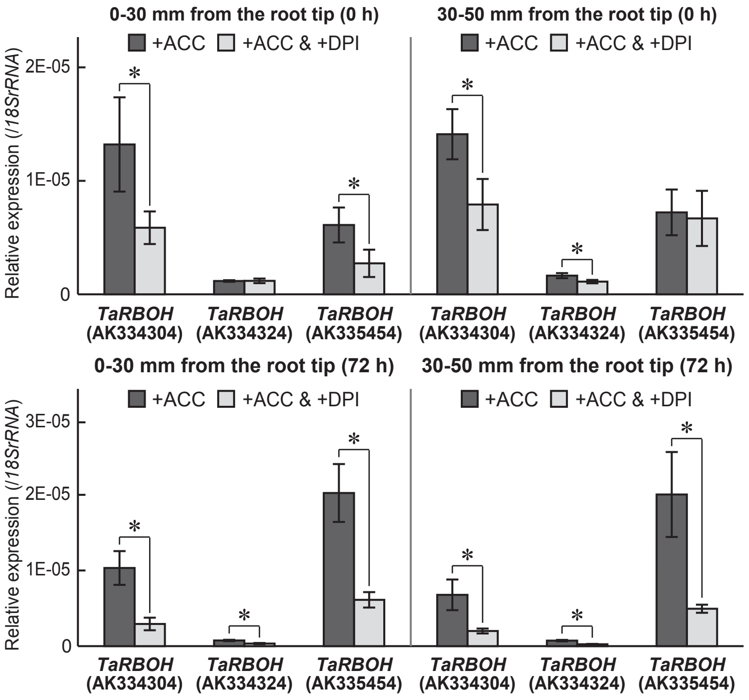

Supplementary Figure S5. Yamauchi et al.

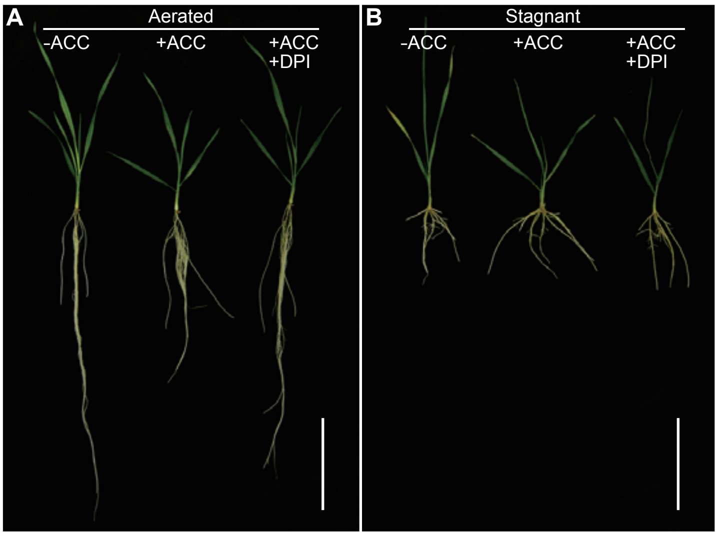

Supplementary Figure S6. Yamauchi et al.

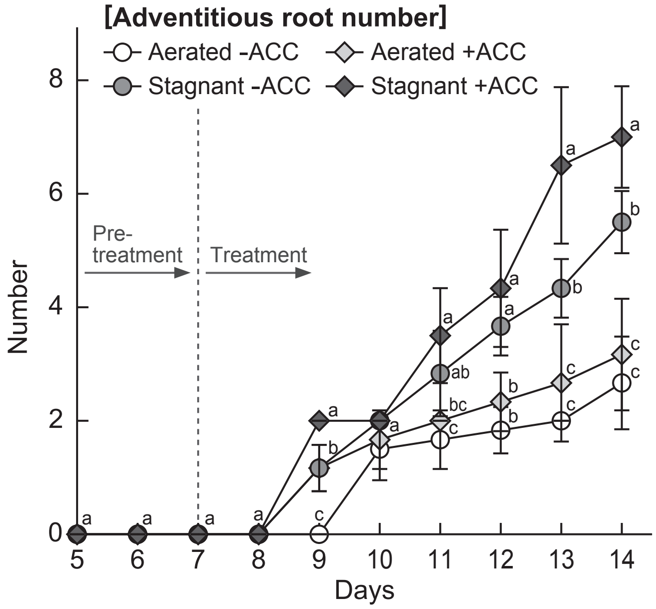

Supplementary Figure S7. Yamauchi et al.

Suppementary Table S1. List of primers for quantitative RT-PCR

Gene Name	NCBI accession	Forward primer sequence	Reverse primer sequence
TaACS2	U42336	5'-AGC ACC AGC AGC AGA AGG-3'	5'-GTG GAC GAG CGG AGA CTG-3'
TaPDC (AK332508)	AK332508	5'-AGG GTC TCT GCT GCC AAC T-3'	5'-TAT TTG ATG GCA ACG TGC TG-3'
TaPDC (BT009420)	BT009420	5'-GAG CTT CTT GAG TGG GGT TCT-3'	5'-ATG TGT CAC TGG GGA TTT GG-3'
TaADH1	EF122847<br>EF122848	5'-CCC AAT GTC GTG GAG ATG TA-3'	5'-CTA GTT CTC CAT GCG GAT GAT-3'
TaADH2	EF122843<br>EF122844<br>EF122845	5'-GGA GCT GGA CGT GGA GAA-3'	5'-GAA CGC CGT GTT GAT CTG-3'
TaADH3	EF122842	5'-GGA GCT GGA GCT GGA GAA-3'	5'-GAG AAG GGC ACG CTA TGC-3'
TaPDH E1α	GU563379	5'-GTT GAG TCA TTT GGC GCA GA-3'	5'-CAT CTT CAT CTT CAG GTT GGT G-3'
TaRBOH (AK334304)	AK334304	5'-GAG GAA GAC GAG CAC CAA A-3'	5'-TTT CCT CGC GAA AAG AGA AA-3'
TaRBOH (AK334324)	AK334324	5'-GTC GGC AGA TTT CAC CCA-3'	5'-CCT GAT GAA GCA TGG CAT AG-3'
TaRBOH (AK335454)	AK335454	5'-CTA ACA AAG GAG CTG CGT GA-3'	5'-AGA AGT TAA AAA TTC TCC TTG TGG-3'
Ta18SrRNA	M82356	5'-GTG ACG GGT GAC GGA GAA TT-3'	5'-GAC ACT AAT GCG CCC GGT AT-3'

Supplementary Table S2. Growth of wheat seedlings under aerated conditions with or without ACC and DPI treatments

Conditions	Before treatments (day 5)		After treatments (day 7)					
	Length (mm)		Length (mm)		Elongation (mm)		Number	
	Shoot	First seminal root	Shoot	First seminal root	Shoot	First seminal root	Leaf	Seminal root
Aerated -ACC	64.2 ± 2.79[a]	60.1 ± 4.69[a]	118.1 ± 4.21[a]	117.9 ± 5.62[a]	53.9 ± 3.95[a]	57.8 ± 4.29[a]	2.0 ± 0.00	5.0 ± 0.00
Aerated +ACC	64.2 ± 2.98[a]	62.3 ± 3.50[a]	107.3 ± 5.14[b]	89.1 ± 4.10[b]	43.4 ± 3.57[b]	29.0 ± 3.07[b]	2.0 ± 0.00	5.0 ± 0.00
Aerated +ACC +DPI	63.9 ± 3.71[a]	60.1 ± 4.10[a]	105.2 ± 3.08[b]	93.4 ± 5.19[b]	40.9 ± 2.88[b]	31.1 ± 3.39[b]	2.0 ± 0.00	5.0 ± 0.00

Plants were grown in aerated conditions with or without 20 µM ACC and 0.1 µM DPI for 2 days. All values are means (n = 18) ±SD. Different lower-case letters denote significant differences among each growth paramater ($P < 0.05$, one-way ANOVA and then Tukey's test for multiple comparisons).
